# Optimization of eIF4E-Binding Peptide Pep8 to Disrupt the RBM38-eIF4E Complex for Induction of p53 and Tumor Suppression

**DOI:** 10.3389/fonc.2022.893062

**Published:** 2022-04-28

**Authors:** Christopher A. Lucchesi, Jin Zhang, Demitria M. Vasilatis, Elizabeth Yip, Xinbin Chen

**Affiliations:** Comparative Oncology Laboratory, Schools of Veterinary Medicine and Medicine, University of California at Davis, Davis, CA, United States

**Keywords:** eIF4E, RBM38, p53, eIF4E-binding peptide, peptide, radiotherapy

## Abstract

Interaction of RNA-binding protein RBM38 with eIF4E on *p53* mRNA is known to suppress *p53* mRNA translation, which can be disrupted by an 8-amino acid peptide (Pep8-YPYAASPA) derived from RBM38, leading to induction of p53 and tumor suppression. Here, we rationally designed multiple Pep8 derivatives and screened for their binding affinities towards eIF4E *in silico.* We showed that several key residues within Pep8 are necessary for its structure and function. We identified a shortened 7-amino acid peptide (Pep7-PSAASPV) that has the highest affinity towards eIF4E and is the most potent inducer of p53 expression. We found that iRGD is an effective vehicle to deliver Pep7 inside of cells for induction of p53 expression and growth suppression as compared to other cell penetrating peptides (Penetratin and Pep-1). We found that peptide cyclization enhances Pep8 affinity for eIF4E, induction of p53 and tumor cell growth suppression. We also found that the ability of Pep7 to induce p53 expression and growth suppression is conserved in cells derived from canine osteosarcoma, a spontaneous tumor model frequently used for testing the feasibility of a therapeutic agent for human cancer. Moreover, we showed that both human and canine osteosarcoma cells, which are notoriously resistant to radiation therapy, were sensitized by Pep7 to radiation-induced growth suppression and cell death. Together, our data suggest that Pep7 may be explored to sensitize tumors to radiation therapy.

## Introduction

A mounting number of studies indicate that therapeutic peptides may be of vast benefit for drug discovery and development in the treatment of malignancies (1). Peptides have several advantages over small molecules inhibitors, including minimal immunogenicity, low toxicity, high target selectivity and affinity, and low cost manufacturability (2). However, there are drawbacks related to decreased bioactivities due to instability and poor tumor penetrability (1). One of the biggest hurdles associated with the use of peptides as drugs that have intracellular targets is how to transport these peptides across the cell membrane. Starting in 1988 with the discovery of the first cell penetrating peptide (CPP), transactivator of transcription (TAT) derived from the human immunodeficiency virus, many different CPPs have been developed to facilitate the delivery of different cargos across the cell membrane (3). There are multiple classes of CPPs, including cationic (Penetratin, TAT, Polyarginine), amphipathic (Pep-1, MPG, pVEC), hydrophobic (Pep-7, Bip, Pept1), and anionic [Map12, SAP(E)]. CPPs competently transport their cargo intracellularly, however, CPPs cannot distinguish between healthy and malignant cells (4). To circumvent this issue, multiple tumor homing peptides have been developed, including iRGD, which directly targets tumor cells (5).

The p53 transcription factor is a crucial tumor suppressor and leading regulator of numerous signaling pathways involved in all aspects of tumor suppression. Functionally, activated p53 stimulates multiple antiproliferative mechanisms by modulating expression of genes involved in DNA repair, cell cycle arrest, apoptosis, and senescence (6). A hallmark and driver of tumor progression is the loss of wild-type p53 function. Like its human counterpart, canine p53 is also frequently altered in various types of cancers. Pharmacological activation of wild-type p53 by targeting proteins which inhibit p53 function/expression is a promising therapeutic approach for malignancies with wild-type p53 in human and canines alike. For example, ALRN-6924, an alpha-helical p53-stapled peptide, was designed to inhibit the binding of two potent p53-inhibitors, MDMX and MDM2, to the p53 tumor suppressor protein. ALRN-6924 has shown promising anti-tumor activity and is currently in phase I and II clinical testing in solid tumors and lymphomas. These positive clinical data from ALRN-6924 have also encouraged the development of peptide-based drugs to activate wild-type p53, such as Pep8, an eight amino acid peptide (YPYAASPA) (7).

Our group previously discovered that RBM38 inhibits *p53* translation *via* interacting with eIF4E and *p53* 3′-UTR, effectively preventing eIF4E from binding to *p53* m^7^G cap halting its translation (8). Of importance, therapeutically targeting eIF4E with Pep8 was found to abrogate the RBM38-eIF4E complex, induce wild-type p53 expression, and sensitize cancer cells to doxorubicin, *in vitro* and *in vivo* (7). While the human and canine RBM38 gene share 96% sequence homology, the Pep8 derived sequence is identical. Additionally, canine and human eIF4E, the target for Pep8, share 99.5% sequence homology, prompting us to hypothesize that Pep8 may also be used to enhance wild-type p53 expression in canine malignancies as well as human. We ultimately discovered that a Pep8 derivative, Pep7, was the most potent inducer of wild-type p53 expression in human and canine malignances, and further, leads to radiosensitivity in both human and canine osteosarcoma (OSA) cell lines.

## Materials and Methods

### Cell Lines

RKO, MCF7 and HCT116 cell lines were cultured at 37°C in DMEM (Gibco 11875085, Fisher Scientific) supplemented with 10% fetal bovine serum (Hyclone, Logan, UT, USA) in a humidified incubator with 5% CO_2_. Human osteosarcoma cell line SJSA1 and canine osteosarcoma cell lines Gracie and D17 were cultured at 37°C in RMPI 1640 (Dulbecco’s Modified Eagle’s medium, Invitrogen) supplemented with 10% fetal bovine serum (Hyclone, Logan, UT, USA) in a humidified incubator with 5% CO_2_. Cell lines were used below passage 25 or within 2 months after thawing. Cells were tested negative for mycoplasma after thawing.

### Cell Line Generation

Generation of the RBM24 and RBM38 double knockout cell line was performed by knocking out RBM24 in RBM38-null cell lines as previously described (7). Briefly, RBM24 knockout cell lines were generated by CRISPR-cas9 genome editing method. sgRNAs targeting RBM24 were designed using the CRISPR design tool and cloned into the BbsI sites of CRISPR vector pSpCas9(BB)-2A-Puro. Two specific gRNAs were used: gRNA #1 GTA CAC CAA GAT CTT CGT CG and gRNA #2 CGA GGT CTT CGG CGA GAT CG. The cells were selected with puromycin and each individual clone was confirmed by western blot and sequencing analysis. Generation of HCT116 ΔC17/− was as previously described (9).

### Plasmids Generation

GST-RBM38 expression plasmid was generated as previously described (10). pTXB1-eIF4E plasmid was generated by amplifying eIF4E using His-eIF4E expression plasmid as template (7). The amplicon was then cloned into pTXB1 *via* NdeI and SapI. The primers used to amplify eIF4E were forward primer, 5´- GGT CAT ATG GCG ACT GTC GAA CCG GAA ACC −3´, and reverse primer, 5´- GGT TGC TCT TCC GCA AAC AAA CCT ATT TTT AGT GGT GGA G −3´.

### Western Blot Analysis and Immunoprecipitation-Western Blot Analysis

Western blot procedures were as previously described (11). Briefly, cell lysates were resolved in an 8-12% SDS-polyacrylamide gel and then transferred to nitrocellulose membranes. Blots were blocked in PBST containing 3% milk for 1 hour at 20°C. Primary antibodies in PBST containing 3% milk were incubated at 4°C rocking overnight. The following morning, membranes were washed 3x with PBST followed by the addition of secondary antibody in PBST containing 3% milk at 20°C for 2 hours. Membranes were then washed 3x with PBST.

### Competitive Pull-Down Assays

For pTXB1-eIF4E, protein expression and purification was as previously described (12). For GST-RBM38 competitive pull-down assays, pGEX-4T3-RBM38 plasmid was transformed into BL21 (DE3) competent E. coli. One-liter culture was grown at 37°C until OD600 equaled 0.6-0.8 and then was induced with a final concentration of 0.1 mM IPTG for 4 hours. Bacteria were pelleted and placed in -80°C overnight. Pellets were then lysed, sonicated, and centrifuged in 20 mL lysis buffer (50 mM Tris pH 7.5, 150 mM NaCl and 0.1% Triton X-100) supplemented with benzonase, 1 mM DTT and protease inhibitor cocktail. Lysates were then incubated with GST beads rocking at 4°C for 2 hours. Beads were washed 3x with lysis buffer. After brief centrifugation, lysates were carefully removed. Beads were then resuspended in lysis buffer with 0.1% Triton X-100 to make a 50% bead slurry. One-hundred microliter bead slurry was incubated in 830 µL lysis buffer, 10 µM (20 µL) peptide (Ctrl, Pep8-1, Pep7_S2K, Pep7, or Pep8_SK) and 250 µg (50 µL) purified eIF4E in a 1.5 mL tube. Samples were rocked overnight, washed 3x with lysis buffer and eluted with 60 µL of 1x SDS-loading buffer before western blot analysis. For eIF4E-eIF4G competitive pulldowns, two 10 cm plates were seeded (1x10^6 RKO cells) for each control and Pep7 treated groups. The next day the cells were lysed with 1 mL lysis buffer (50 mM Tris pH 7.5, 150 mM NaCl and 0.1% Triton X-100) supplemented with benzonase, 1 mM DTT and protease inhibitor cocktail. After brief sonication (5x, 5 seconds on, 15 seconds off), lysates were centrifuged, and then added to a fresh tube. Magnetic protein A/G beads (30 µL) were added to each tube followed by 1 µg IgG or eIF4E antibody. After 1 hour 25 µM control or Pep7 peptides were added to the corresponding group. After rotating overnight at 4°C, beads were washed 5x with lysis buffer before western blot analysis.

### 2-D Cell Viability Assay

For 2-D cell viability assays, 15,000 cells per well were plated in a triplicate in a 96 well plate. Two hours later, 20 µM peptide was added to each well. Twenty-four hours later, cell viability was measured by CellTiter-Glo 3D according to manufacturer’s guidelines (Promega).

### 3-D Tumor Sphere Assays

3-D mini ring culture assays were as described previously (13). Briefly, single cell suspensions (15K cells/well) were plated around the rim of the well in 96-well plates in a 4:3 mixture of Matrigel and Mammocult (BD Bioscience CB-40324). After plates were incubated at 37°C with 5% CO_2_ for 15 min to solidify the gel, 100 µL of prewarmed Mammocult containing the indicated peptide was added to the corresponding well. Four hours after peptide addition, the irradiated sample group was irradiated with 15 Gy radiation. Twenty-four hours after irradiation (or no irradiation) the media was replaced with 100 µL of prewarmed Mammocult. Seventy-two hrs later, 100 µL pre-warmed PBS was used to wash the cells 3 times. Cells were then released from the Matrigel by incubating at 37°C for 45 min in 50 μL of 5 mg/mL dispase (Life Technologies #17105-041). Images were taken with a 40x objective and then cell viability was measured by CellTiter-Glo 3D according to manufacturer’s guidelines (Promega). The 3-D breast cancer spheroid cultures were as described previously (7). Briefly, MCF7 cells were cultured in MammoCult™ (Stemcell, Cambridge, MA) media per manufacturer’s guidelines. Twenty-thousand cells per well were seeded in 6-well ultra-low adherent plates. The following day the cells were treated with 5 μM Pen-Ctrl or Pen-Pep8. After 7 days, tumor spheres larger than 50 μm were counted.

### LC50 Determination

Cells were seeded in 96-well plates (~7,000 cells per well) and were incubated at 37°C, 5% CO_2_ for 24 h. After the incubation period, cells were exposed to a range of drug concentrations in triplicates, and were incubated in the same conditions. After 48 h exposure, cell viability was measured using CellTiter-Glo 3D according to manufacturer’s guidelines (Promega). We estimated LC50 values using a logistic regression (% cell death) model.

### Peptide Docking Simulations

The docking program AutoDock Vina (14), within the SAMSON-Connect interface (https://www.samson-connect.net/) was used to model the docking of each peptide to eIF4E. First, Pep-FOLD3 was used to determine the estimated secondary structure of each peptide (15). Next, the experimental X-ray crystal structure of eIF4E (with water removed) was used for the peptide docking assays (PDB code: 1WKW). A grid box was drawn 5 Å around the previously identified binding site for Pep8 (7). Each peptide candidate had missing hydrogens added and where energy minimized. AutoDock Vina was set to an exhaustiveness of 8 and the top 5 modes were used to calculate the average affinity (kcal/mol) and Ki (umol).

## Materials

Peptides were synthesized by GenScript (Piscataway, NJ). Antibodies used were: anti-p53 (1C12, Cell Signaling, Danvers, MA), anti-Actin (Sigma), anti-GST (B-14, Santa Cruz, Dallas, TX), anti-eIF4E (P-2, Santa Cruz, Dallas, TX), anti-Vinculin (7F9, Santa Cruz, Dallas, TX), and anti-phospho-histone H2A.X (Ser139) (20E3, Cell Signaling, Danvers, MA)

### Statistical Analysis

Experimental values are presented as mean ± SEM. Statistical comparisons between experimental groups were analyzed by a two-tailed Student’s t-test. P values < 0.05 were considered statistically significant.

## Results

### Delineating Key Residues in Pep8 Necessary for Binding to eIF4E

**Figure 1:**
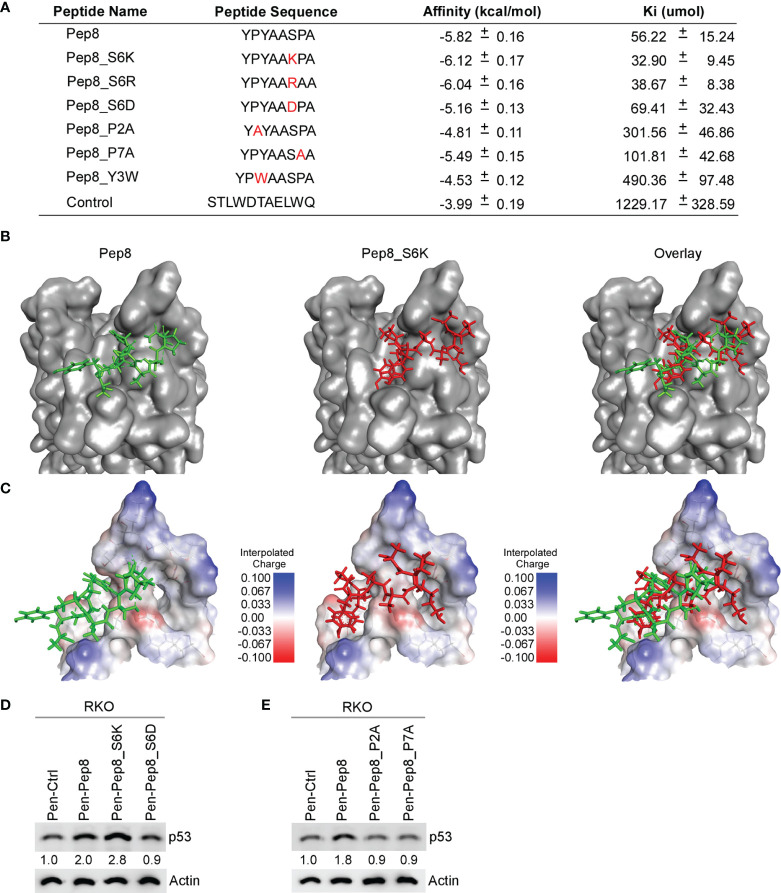
Delineating key residues in Pep8 necessary for binding to eIF4E. **(A)** List of peptide names and sequences, as well as the calculated binding affinity and equilibrium dissociation constant (Ki) for each peptide as determined by AutoDock Vina. **(B)** Visualization of the binding mode for Pep8 and Pep8_S6K. **(C)** Visualization of the interpolated charge surface of eIF4E, and the corresponding peptide. **(D, E)** The levels of p53 and actin proteins were measured in RKO cells treated with 20 µM Penetratin fused peptides for 18 hrs.

Previously, we identified Pep8, an 8-amino acid peptide derived from the carboxyl-terminus of RBM38 that enhances *p53* translation by disrupting the RBM38-eIF4E complex leading to tumor growth suppression *in vitro* and *in vivo* (7). Further, we determined that Ser:6 of Pep8, which is necessary for its interaction with eIF4E, forms a hydrogen bond with Asp:202 of eIF4E (7). In an effort to enhance the affinity of Pep8 for eIF4E, we performed *in-silico* docking studies on rationally designed Pep8 derivatives. Based on replica exchange molecular dynamic simulations (REMDS) (7), multiple Pep8 derivatives were predicted to have either enhanced or decreased affinity towards eIF4E (**Figure 1A**). First, we determined the secondary structures of each peptide using PEP-FOLD3. PEP-FOLD3 is a novel computational framework, which allows for *de novo* free or biased prediction for linear peptides between 5 and 50 amino acids (15). Second, AutoDock Vina (14), an *in silico* docking program, was used to determine the affinity of each peptide for eIF4E. **Figure 1A** summarizes the peptide sequence, binding affinity, and Ki of each peptide tested. Since Ser:6 in Pep8 forms a hydrogen bond with Asp:202 in eIF4E, we hypothesized that substituting Ser:6 with a positive charge (Ser to Lys or Arg, Pep8_S6K and Pep8_S6R) would enhance its affinity towards eIF4E, while substituting to a negative charge (Ser to Asp, Pep8_S6D) should decrease its affinity*. In silico* docking predicted that Pep8_S6R and Pep8_S6K have higher affinities for eIF4E, whereas, Pep8_S6D was predicted to have a lower affinity for eIF4E than Pep8 (**Figure 1A**). Furthermore, because prolines are known to modulate or define the structure of a peptide (16), we asked whether both prolines are necessary for Pep8 function. Docking analysis revealed that both Pep8_P2A and Pep8_P7A have less affinity towards eIF4E than Pep8 (**Figure 1A**), demonstrating that these proline residues may be necessary for Pep8 structure and/or function. Moreover, docking analysis showed that Pep8_Y3W has little affinity towards eIF4E, suggesting that Tyr:3 in Pep8 may also be important for peptide structure and/or binding.

Our *in silico* docking assays demonstrated two differential docking patterns for Pep8 and Pep8 S6K (**Figures 1B, C**). Importantly, Lys:6 in Pep8 S6K formed a tighter hydrogen bond with Asp:202 in eIF4E than did Ser:6 in Pep8 (3.0 Å vs 3.7 Å). To confirm our docking results, western blot analysis was performed to measure induction of wild-type p53 in RKO colon cancer cells treated with these peptide derivatives. First, we tested whether Pep8_S6K would enhance p53 induction, whereas Pep8_S6D would be inert. In order to facilitate intracellular delivery, the cell-penetrating peptide Penetratin was fused to these peptides at their N-termini (17). As shown in **Figure 1D**, Pep8_S6K was more potent than Pep8 to induce p53 expression, whereas Pep8_S6D was inactive. In addition, both prolines in Pep8 were necessary for its function as highlighted by lack of p53 induction in cells treated with either Pep8_P2A or Pep8_P7A (**Figure 1E**).

### Identification of Pep7, Which has the Highest Affinity Towards eIF4E and is the Most Potent Inducer of p53 Expression

**Figure 2:**
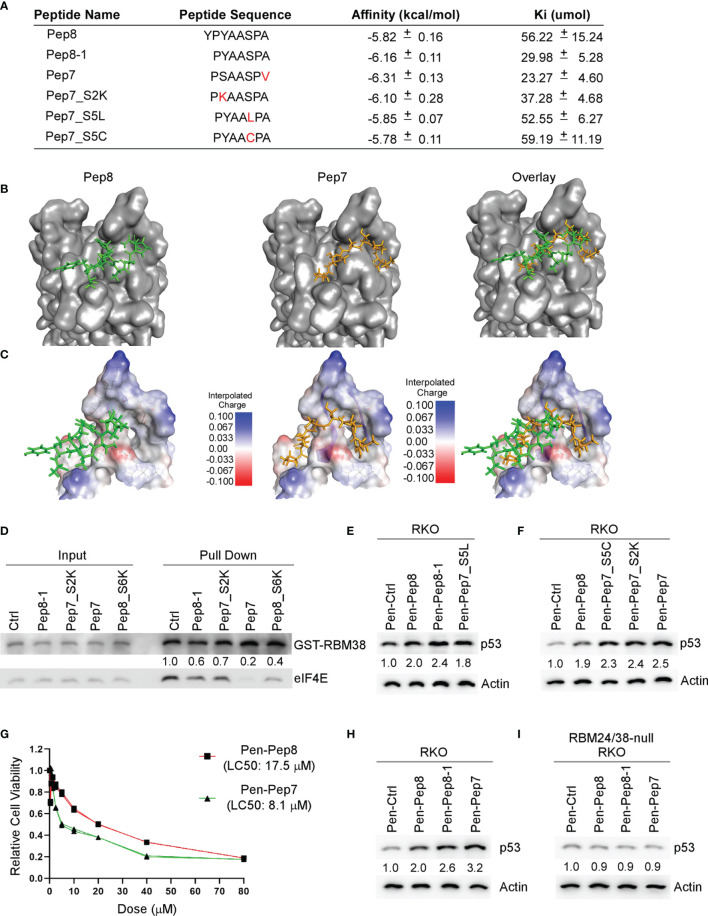
Bioactivity optimization of Pep8 peptide. **(A)** List of peptide names and sequences, as well as the calculated binding affinity and equilibrium dissociation constant (Ki) for each peptide as determined by AutoDock Vina. **(B)** Visualization of the binding mode for Pep8 and Pep7. **(C)** Visualization of the interpolated charge surface of eIF4E and the corresponding peptide. **(D)** Immunoblot for the competitive pull-down assay for GST-RBM38 and eIF4E with the addition of Pep8 derivatives. **(E, F)** The levels of p53 and actin proteins were measured in RKO cells treated with 20 µM Penetratin fused peptides for 18 hrs. **(G)** RKO colon cancer cells were exposed (in triplicates) to a wide range of concentrations of either Pen-Pep8 or Pen-Pep7 (0.3125 – 80 µM) and the lethal concentration for 50% cell death (LC50) was calculated based on a sigmoidal effect (% cell death) model. **(H, I)** The levels of p53 and actin proteins were measured in wild-type **(H)** and RBM24/RBM38 double knockout **(I)** RKO cells treated with 20 µM Penetratin fused peptides for 18 hrs.

Our initial Pep8 derivative docking studies revealed that the first tyrosine in Pep8 was not necessary for Pep8 to bind with eIF4E, therefore, we generated five rationally designed 7-amino acid peptide derivatives (**Figure 2A**). Our *in silico* docking results revealed that all Pep7 derivatives, except for Pep7_S5C, had higher affinities for eIF4E than Pep8, whereas Pep7 was shown to have the highest affinity of all the peptides tested (**Figure 2A**). Further, the docking analysis revealed a differential docking pattern for Pep7 when compared to Pep8 (**Figures 2B, C**). We postulate that while only 3 out of 8 residues in Pep8 (Tyr:3, Ser:6, and Ala:8) form hydrogen bonds with eIF4E, 6 out of 7 residues in Pep7 (Pro:1, Ser:2, Ala:3, Ala:4. Ser:5, and Pro:6) form hydrogen bonds with eIF4E, which likely contribute to its elevated affinity towards eIF4E.

As Pep7 derivatives were shown to have the highest affinities towards eIF4E (**Figure 2A**), we questioned whether Pep7 and its derivates were able to modulate the interaction between RBM38 and eIF4E. To that end, we performed competitive pull-down assays with purified GST-tagged RBM38 and purified eIF4E in the presence of control or Pep8-derived peptides. We found that these Pep8 derivatives were able to impede the interaction between RBM38 and eIF4E, with Pep7 exhibiting the strongest activity (**Figure 2D**).

To confirm the docking results, western blot analysis was performed to measure p53 induction in RKO colon cancer cells treated with these peptide derivatives. We found that these Pep8 derivatives induce p53 expression equal to or greater than Pep8, with Pep7 being the strongest, consistent with our *in silico* docking results (**Figures 2E, F**). To determine if Pep7 is more efficacious than Pep8, we calculated the LC50 for both Pep8 and Pep7 in RKO cells. As shown in **Figure 2G**, Pep7 was more than twice as potent (LC50: 8.1 µM) as Pep8 (LC50: 17.5 µM), further confirming our aforementioned results.

To define the specificity of Pep7 to induce p53 by disruption of the RBM24/RBM38-eIF4E complex, p53 induction was measured in an isogenic control and RBM24/RBM38 double knockout RKO cells treated with Pen-Ctrl, Pen-Pep7, Pen-Pep8, or Pen-Pep8-1. We showed similar to Pep8 and Pep8-1, Pep7 was able to induce p53 expression in wild-type but not double knockout cells (**Figures 2H, I**). To determine whether Pep7 induces p53 expression through binding to eIF4E, the effect of these Pep8 derivatives on p53 induction was measured in isogenic control and eIF4E-deficient HCT116 colon cancer cells (ΔC17/−). Previously, we showed that the ΔC17/− HCT116 cell line has only one truncated eIF4E allele, in which the last 17 residues, including Asp:202, are deleted, abrogating the ability of RBM38 to interact with eIF4E (9). Similar to Pep8 and Pep8-1, Pep7 was able to induce p53 expression in isogenic control, but not eIF4E-deficient HCT116 cells (**Supplementary Figures 1A, B**). Likewise, Pep7 was able to decrease tumor cell viability in wild-type but not in ΔC17/− HCT116 cells (**Supplementary Figures 1C**). To rule out the possibility that Pep7 inhibits eIF4G ability to interact with eIF4E, we performed a competitive pulldown assay with the addition of Pep7. We showed that Pep7 was unable to impede the interaction between eIF4E and eIF4G (**Supplementary Figure 1D**). In addition, we modeled the binding of Pep7 to eIF4E in the presence of eIF4G or 4E-BP1 (the inhibitor of eIF4G), and determined based on its binding interface, it is unlikely that Pep7 is able to modulate the interaction of eIF4G or 4E-BP1 with eIF4E (**Supplementary Figures 1E, F**). Collectively, these data indicate that Pep7 is a specific inhibitor of the RBM38-eIF4E complex and is the most potent inducer of p53 expression through eIF4E- and RBM24/RBM38-dependent manners.

### Cyclization of Pep8 Enhances Induction of p53 and Growth Suppression

**Figure 3:**
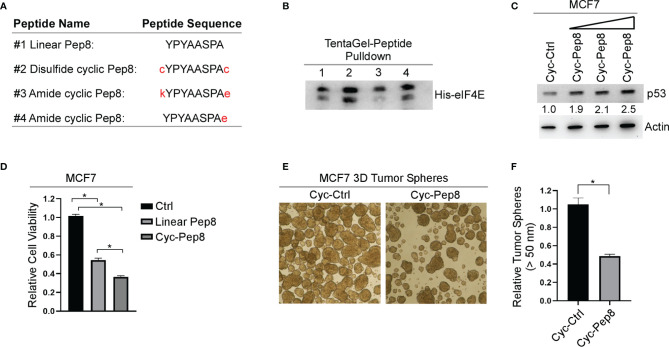
Cyclized Pep8 enhances p53 expression. **(A)** Sequence of each peptide used and mode of cyclization. **(B)** Immunoblot for the pull-down of eIF4E with TentaGel-bound peptides. **(C)** The levels of p53 and actin were measured in MCF7 cells treated with increasing concentrations of Pep-1 delivered disulfide cyclized Pep8 (50 nM, 150 nM, and 375 nM) for 18 hrs. **(D)** Relative cell viability was measured in MCF7 2-D cell cultures treated with Pep-1 delivered control, linear-Pep8 or disulfide cyclic-Pep8 (375 nM) peptide for 24 hrs. Values represent the mean ± SEM of three independent experiments (*, P < 0.05). **(E, F)** Disulfide cyclized Pep8 (375 nM) treatment reduces MCF7 tumor sphere formation. Tumor spheres (>50 mm) counted 7 days after peptide treatment. Values represent the mean ± SEM of three independent experiments (*, P < 0.05).

Constraining a peptide *via* cyclization is a frequently used strategy for maintaining the secondary structure of the peptide, and for enhancing conformational stability (18). Therefore, we generated three different cyclic Pep8 peptides using two methods of cyclization. First, disulfide cyclization was utilized by flanking Pep8 with two cystine residues (cYPYAASPAc, referred to as cyclic Pep8). Second, amide bond cyclization was performed by flanking Pep8 with a lysine and glutamic acid (kYPYAASPAe), or by a single glutamic acid on its C-terminus (YPYAASPAe) (**Figure 3A**). To delineate whether these peptides were able to interact with eIF4E, both linear Pep8 and cyclic peptides were conjugated to TentaGel resins for pulldown assays. We showed that all four peptides interacted with eIF4E, with cyclic Pep8 exhibiting the strongest interaction (**Figure 3B**). Next, we asked whether cyclic Pep8 is able to induce p53 expression. To facilitate intracellular delivery of cyclic Pep8, we used Pep-1 CPP which is a short amphipathic peptide carrier which releases its cargo after intracellular delivery (19). As shown in **Figure 3C**, cyclic Pep8 was able to induce p53 expression in a dose-dependent manner (50 nM, 150 nM, and 375 nM). To determine if cyclic-Pep8 is more potent than linear Pep8, we performed a cell viability assay in MCF7 cells treated with Pep-1 delivered control, linear Pep8 and or cyclic Pep8 peptides. We found that cyclic Pep8 was more potent than linear Pep8 to suppress tumor cell growth (**Figure 3D**). Further, we demonstrated that cyclic Pep8 significantly decreased the formation of MCF7 3D tumor spheres (**Figures 3E, F**). Together, these results demonstrate that disulfide cyclized Pep8 is a potent inducer of p53 and suppressor of 3D tumor sphere formation.

### iRGD Is an Effective Vehicle to Deliver Pep7 Inside of Cells to Induce p53 Expression and Growth Suppression

**Figure 4:**
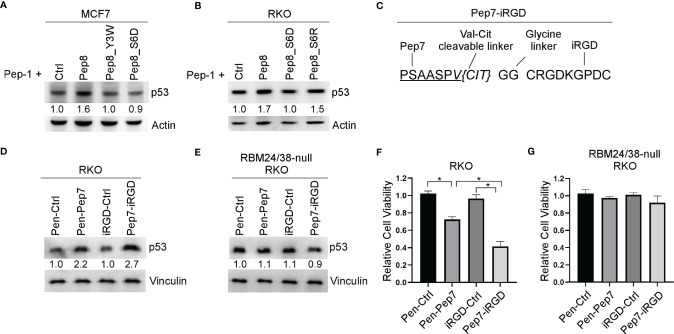
iRGD is the optimal CPP for intracellular delivery of Pep7. **(A, B)** The levels of p53 and actin proteins were measured in MCF7 **(A)** or RKO **(B)** cells treated with Pep-1 delivered peptides (375 nM) for 18 hrs. **(C)** Peptide sequence for iRGD-conjugated Pep7. **(D, E)** The levels of p53 and vinculin were measured in wild-type **(D)** and RBM24/RBM38 double knockout **(E)** RKO cells treated with Penetratin or iRGD delivered peptides (20 µM) for 18 hrs. **(F, G)** Relative cell viability was measured in wild-type **(F)** and RBM24/RBM38 double knockout **(G)** RKO 2-D cell cultures treated with indicated peptides for 24 hrs. Values represent the mean ± SEM of three independent experiments (*, P < 0.05).

Even though Penetratin-conjugated control peptide was used as a control, it remains possible that the Penetratin CPP may cooperate with Pep8 to induce p53 expression. To rule out this possibility, Pep8 and its derivatives were delivered into cells by Pep-1 CPP. We showed that p53 expression was induced by Pep8 and Pep8_S6R when delivered by Pep-1, but not Pep8_Y3W and Pep8_S6D (**Figures 4A, B**), suggesting that Pep8 and its derivatives are responsible for inducing p53 expression.

To improve peptide tumor targeting and intracellular delivery, we sought to determine whether iRGD-conjugated Pep7 (Pep7-iRGD) can be efficiently delivered inside of tumor cells (**Figure 4C**). In addition, we coupled Pep7 to iRGD using a valine citrulline (Val-Cit) linker which is cleavable by cathepsin B, which is highly expressed in cancer cells to aid in tumor targeting (20). Next, we measured p53 induction in an isogenic control and RBM24/RBM38 double knockout RKO cells treated with Pen-Pep7 or with Pep7-iRGD. We found that Pep7-iRGD was more competent than Pen-Pep7 to induce p53 expression in isogenic control cells (**Figure 4D**), whereas both Pep7-iRGD and Pen-Pep7 were inert in RBM24/RBM38-null cells (**Figure 4E**). We would like to mention that the fold change of p53 induction by iRGD-Pep7 (2.7 fold) was higher than that by cyclic Pep8 (from 1.9 to 2.5 fold) (**Figure 3C**), indicating that iRGD-conjugated Pep7 is the most potent inducer of p53 and allows for a targeted delivery approach. Similarly, Pep7-iRGD was more competent than Pen-Pep7 to inhibit cell survival in isogenic control cells, but not in RBM24/RBM38-null cells (**Figures 4F, G**). Together, these data support that iRGD is the optimal CPP to deliver Pep7.

### The Ability of Pep7 to Induce p53 Expression and Growth Suppression Is Conserved in Canine Cancer Cells

**Figure 5:**
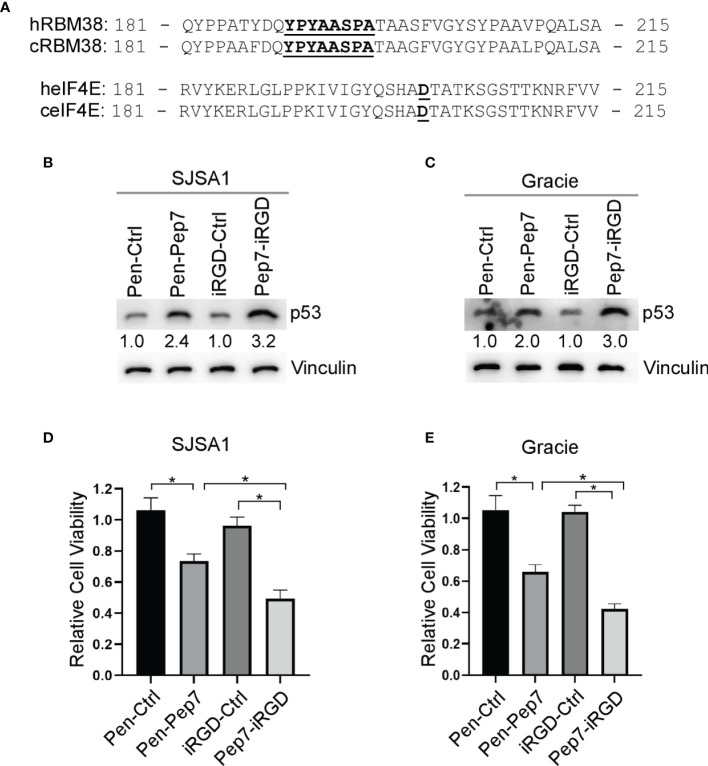
Pep7 enhances p53 and tumor growth suppression in human and canine OSA cells. **(A)** Comparison of human and canine RBM38 and eIF4E protein sequences focused around their respective binding interfaces. **(B, C)** The levels of p53 and vinculin were measured in human OSA (SJSA1) and canine OSA (Gracie) cell lines treated with Penetratin or iRGD delivered peptides (20 µM) for 18 hrs. **(D, E)** Relative cell viability was measured in SJSA1 and Gracie 2-D cell cultures treated with indicated peptides for 24 hrs. Values represent the mean ± SEM of three independent experiments (*, P < 0.05).

While p53 is frequently inactivated in various human and canine tumors, including osteosarcomas (OSA), ~ 60% of OSA contain wild-type p53, thus restoring wild-type p53 function may be an attractive strategy for the treatment of OSA (21). We found that both human and canine RBM38 proteins share ~95% sequence identity, including the region where Pep8 is derived (**Figure 5A**). Further, human and canine eIF4E proteins share ~99% sequence identity (**Figure 5A**). Thus, we hypothesized that Pep7 would be able to induce p53 expression in canine cancer cells. Indeed, p53 expression was induced by Pep7 in human SJSA1 and canine Gracie osteosarcoma cells (**Figures 5B, C**), both of which harbor wild-type p53. Similarly, we found that cell viability was markedly decreased by Pep7 in both cell lines (**Figures 5D, E**). We would like to mention that iRGD-conjugated Pep7 was more potent than Penetratin-conjugated Pep7 to induce p53 expression and growth suppression (**Figures 5B–E**). In addition, Pep8 significantly decreased the cell viability of 3D tumor spheroids from another canine osteosarcoma cell line D17, which expresses wild-type p53 (**Supplementary Figures 2A, B**). These data support our hypotheses that the RBM38-eIF4E pathway is conserved between human and canine and may be of therapeutic relevance.

### Pep7 Is Highly Potent to Sensitize Radio-Resistant Osteosarcoma Cells to Radiation

**Figure 6:**
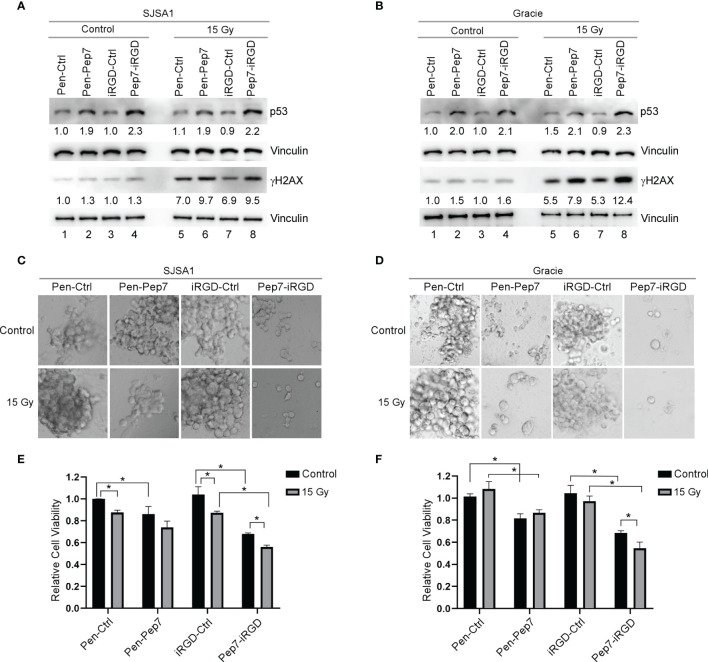
Pep7 sensitizes human and canine OSA cells to radiation. **(A, B)** The levels of p53, γH2AX, and vinculin were measured in human OSA (SJSA1) and canine OSA (Gracie) cell lines treated with Penetratin or iRGD delivered peptides (20 µM) alone or in combination with 15 Gy radiation. **(C–F)** 3D spheroid cultures and relative cell viability were measured in SJSA1 **(C, E)** and Gracie **(D, F)** cells after treatment with peptide alone (20 µM) or in combination with 15 Gy radiation. Spheroids were imaged with a 10x microscope objective. Values represent the mean ± SEM of three independent experiments (*, P < 0.05).

Resistance to chemo-/radio-therapy is a major challenge for long-term survival of cancer patients (22). As induction of wild-type p53 is considered to re-sensitize tumors to chemo-/radio-therapy (23), we postulated that Pep7 may be further explored as a potential cancer therapeutic agent. Previously, we showed that induction of wild-type p53 by Pep8 can sensitize multiple types of tumor cells to chemotherapeutic agents, such as doxorubicin and etoposide (7). Thus, we determined whether induction of p53 by Pep7 can be used to sensitize radio-resistant human SJSA1 and canine Gracie osteosarcoma cells. To test this, SJSA1 and Gracie cells were treated with Penetratin- or iRGD-conjugated Pep7 for 4 hours followed by 15-Gy radiation for 2 hours. As expected, p53 expression was induced in SJSA1 and Gracie cells by both Penetratin- and iRGD-conjugated Pep7 as compared to their control counterparts (**Figures 6A, B**, compare lanes 2 and 4 with 1 and 3, respectively). In contrast, p53 expression was not or only slightly induced 2 hours after 15-Gy radiation (**Figures 6A, B**, compare lane 5 and 7 with 1 and 3 respectively). To confirm that the level of radiation used was sufficient to induce p53 expression, radio-sensitive RKO cells were treated similarly as for SJSA1 and Gracie cells. As expected, p53 expression was induced in RKO cells by Penetratin- and iRGD-conjugated Pep7 as compared to their control counterparts (**Supplementary Figure 3A**, compare lanes 2 and 4 with 1 and 3, respectively). Additionally, we found that p53 expression was highly induced in RKO cells upon 15-Gy radiation (**Supplementary Figure 3A**, compare lanes 5 and 7 with 1 and 3, respectively). These observations suggest that the lack of induction of wild-type p53 in SJSA1 and Gracie cells may play a role in their inherent radio-resistance (24, 25).

To determine whether the dose of radiation was high enough to induce DNA damage, we measured γH2AX, a key indicator of DNA damage (26). We showed that 15-Gy radiation led to a marked increase in the level of γH2AX in SJSA1 and Gracie cells as well as in RKO cells (**Figures 6A, B** and **Supplementary Figure 3A**, compare lane 5 and 7 with 1 and 3, respectively), suggesting that the DNA damage response pathway is functional in SJSA1 and Gracie cells. We also showed that the level of γH2AX was increased in SJSA1, Gracie, and RKO cells treated with Penetratin- and iRGD-conjugated Pep7 as compared to their control counterparts (**Figures 6A, B** and **Supplementary Figure 3A**, compare lanes 6 and 8 with lanes 5 and 7, respectively). These observations are consistent with our previous studies that DNA damage response pathway is activated by Pep8 *via* induction of wild-type p53 (7, 13).

Next, 3D tumor spheroids were established as a model to determine whether Pep7 can sensitize radio-resistant human SJSA1 and canine Gracie osteosarcoma cells to radiation. Radio-sensitive RKO 3D tumor spheroids were established as a control. We showed that Penetratin- or iRGD-conjugated Pep7 was able to suppress tumor spheroid growth in all three cell lines, whereas 15-Gy radiation was only able to suppress radio-sensitive RKO tumor-spheroid growth (**Figures 6C–F** and **Supplementary Figures 3B, C**). However, combined treatment of Pep7-iRGD with radiation made both SJSA1 and Gracie cells highly susceptible to growth suppression and decreased viability (**Figures 6C–F**). Additionally, iRGD-conjugated Pep7 was able to further enhance the sensitivity of RKO cells to radiation (**Supplementary Figures 3B, C**). Collectively, these data support that iRGD-conjugated Pep7 may be used as an adjuvant to sensitize radio-resistant cancer cells to radiation.

## Discussion

It is well understood that restoration of wild-type p53 is an effective strategy to suppress tumor growth in animal models (27–29). Therefore, we sought to enhance the efficacy of Pep8 to disrupt the protein-protein interaction (PPI) between RBM38 and eIF4E using rationally designed peptide derivatives to enhance wild-type *p53* translation as a novel therapeutic approach. Our first generation peptide, Pep8, was shown to abrogate the RBM38-eIF4E complex and enhance wild-type *p53* expression, resulting in the reduced growth of xenograft tumors in nude mice (7). Previously, replica exchange molecular dynamic simulations (REMDS) showed that Pep8 docks with eIF4E *via* a shallow pocket in its carboxyl-terminus, with Ser:6 in Pep8 forming a key hydrogen bond with Asp:202 in eIF4E (7). Armed with this knowledge, Pep8 derivatives were rationally designed to identify key residues within Pep8 necessary for its structure/function to aid in the development of our next generation of peptides. Ultimately, Pep7 was found to be the strongest disruptor of the RBM38-eIF4E complex (**Figure 2D**), inducer of p53 expression, and suppressor of 3D tumor spheroid growth (**Figures 2H, I** and **5D, E**). Because off-target toxicities are often a main reason why drug candidates fail in clinical trials (30), we utilized CRISPR/Cas9 knockout cell lines to validate Pep7 specificity towards abrogating the RBM38-eIF4E complex. Confirming Pep7 specificity, we showed that Pep7 has no effect on p53 expression or cell viability in double knockout RBM24/38 RKO cell lines (**Figures 2H, I** and **4F, G**), nor on eIF4E ΔC17/− HCT116 cells (**Supplementary Figures 1A–C**). In addition, as most eIF4E targeted therapeutic approaches aim to inhibit eIF4E function by either inhibiting its binding to a m^7^G cap or to eIF4G (31), we performed docking studies coupled with pulldown assays to demonstrate that it is unlikely that Pep7 modulates the binding of eIF4G or 4E-BP1 (**Supplementary Figures 1D–F**). Taken together, these assays support that Pep7 is a specific inhibitor of the RBM38-eIF4E complex and potent inducer of wild-type p53.

One of the most challenging aspects of designing a therapeutic peptide with an intracellular target is how to effectively deliver the peptide into the cell. Herein, we tested three different CPPs to facilitate intracellular delivery of our peptide candidates. First, Penetratin was fused to the N-termini of our peptides, which was shown to be effective, as indicated by both increased p53 expression and decreased cell viability in both 2D and 3D cultures (**Figures 4**–**6**). Second, we utilized Pep-1, which forms stable nanoparticles with protein cargo to deliver peptides into the cell. We found that similar to Penetratin, Pep-1-delivered peptides were able to induce p53 expression (**Figures 3C**, **4A, B**). Moreover, Pep-1 delivered disulfide bond cyclized Pep8 was found to be more potent than linear Pep-1 delivered Pep8 (**Figure 3D**). However, like Penetratin, Pep-1 is not tumor targeting which may cause off target toxicities or decreased bioavailability, and further, the Pep-1 based system has a limitation that the efficiency of transduction is linked to the particle size and the cellular uptake mechanism (19), both of which are hard to control. Therefore, for our third CPP we used iRGD with a cathepsin-B cleavable valine-citrulline linker to mitigate the shortcomings with both Penetratin and Pep-1 based CPP approaches. iRGD has three distinct sites contributing to its tumor targeting and cell-penetrating abilities (**Figure 3C**): a tumor homing motif, a C-end Rule (CendR) penetration motif, and a proteolytic cleavage recognition site (32). Furthermore, we used a valine citrulline (Val-Cit) linker which is cleavable by cathepsin B, a protease highly expressed in cancer cells (20). This approach allows for targeted delivery (unlike both Penetratin and Pep-1) and for the peptide to be free from its carrier (unlike Penetratin). Ultimately, we found that iRGD-conjugated Pep7 was highly effective at inducing p53 expression and suppressing both 2D and 3D tumor cultures (**Figures 4D–G**, **5**, **6**).

The incidence rate for OSA in canines is 27 times higher than in humans and canine OSA has been used as a viable comparative model for human OSA (33). Using the NCBI database, we found that both human and canine RBM38 and eIF4E genes share a high sequence identify (95% and 99%, respectively). Since ~ 60% of OSAs contain wild-type p53 (21), we hypothesized that Pep7 may be used to suppress human and canine OSA cell growth *via* induction of wild-type p53. Indeed, both Penetratin- and iRGD- conjugated Pep7 peptides were able to induce p53 expression and suppress tumor cell growth (**Figure 5**). Since both canine and human OSAs are known to be highly radio-resistant, we tested whether Pep7 can be used to sensitize human and canine OSA cancer cells to radiation, a key therapeutic strategy for cancer treatment. We confirmed that both human and canine OSA cell lines have little or no radio-sensitivity (**Figures 6E, F**). Interestingly, we found that p53 expression in both OSA cell lines did not respond to radiotherapy, potentially highlighting one of the reasons for their radio-resistance. With therapeutic importance, we found that the sensitivity of both human and canine OSA cells to radiation was markedly increased by iRGD delivered Pep7, possibly due to the increased expression of p53 in the Pep7 treated cells.

## Conclusion

In summary, we rationally designed multiple Pep8 derivatives and identified Pep7 as the most potent inducer of p53 expression and tumor suppression. We also found that iRGD delivered Pep7 is highly potent to sensitize both human and canine OSA cells to radiotherapy. Our studies suggest that iRGD-conjugated Pep7 may be explored as an adjuvant agent to sensitize radio-resistant tumors to radiotherapy.

## Data Availability Statement

The raw data supporting the conclusions of this article will be made available by the authors, without undue reservation.

## Author Contributions

The authors confirm contribution to the paper as follows: study conception and design: CAL, XC; data collection: CAL, DMV, EY; analysis and interpretation of results: CAL, JZ, XC; draft manuscript preparation: CAL, JZ, XC. All authors reviewed the results and approved the final version of the manuscript.

## Funding

NIH: RO1 CA250338 NIH: RO1 CA081237 HHS: HHSN261200800001E NIH:T32 CA108459 NIH: T32 OD01147

## Conflict of Interest

The authors declare that the research was conducted in the absence of any commercial or financial relationships that could be construed as a potential conflict of interest.

## Publisher’s Note

All claims expressed in this article are solely those of the authors and do not necessarily represent those of their affiliated organizations, or those of the publisher, the editors and the reviewers. Any product that may be evaluated in this article, or claim that may be made by its manufacturer, is not guaranteed or endorsed by the publisher.
