# Supplementary figures and images for: Optimization of eIF4E-Binding Peptide Pep8 to Disrupt the RBM38-eIF4E Complex for Induction of p53 and Tumor Suppression
Source: Front Oncol. 2022 Apr 28;12:893062. doi: 10.3389/fonc.2022.893062 (PMC9095979; doi:10.3389/fonc.2022.893062)

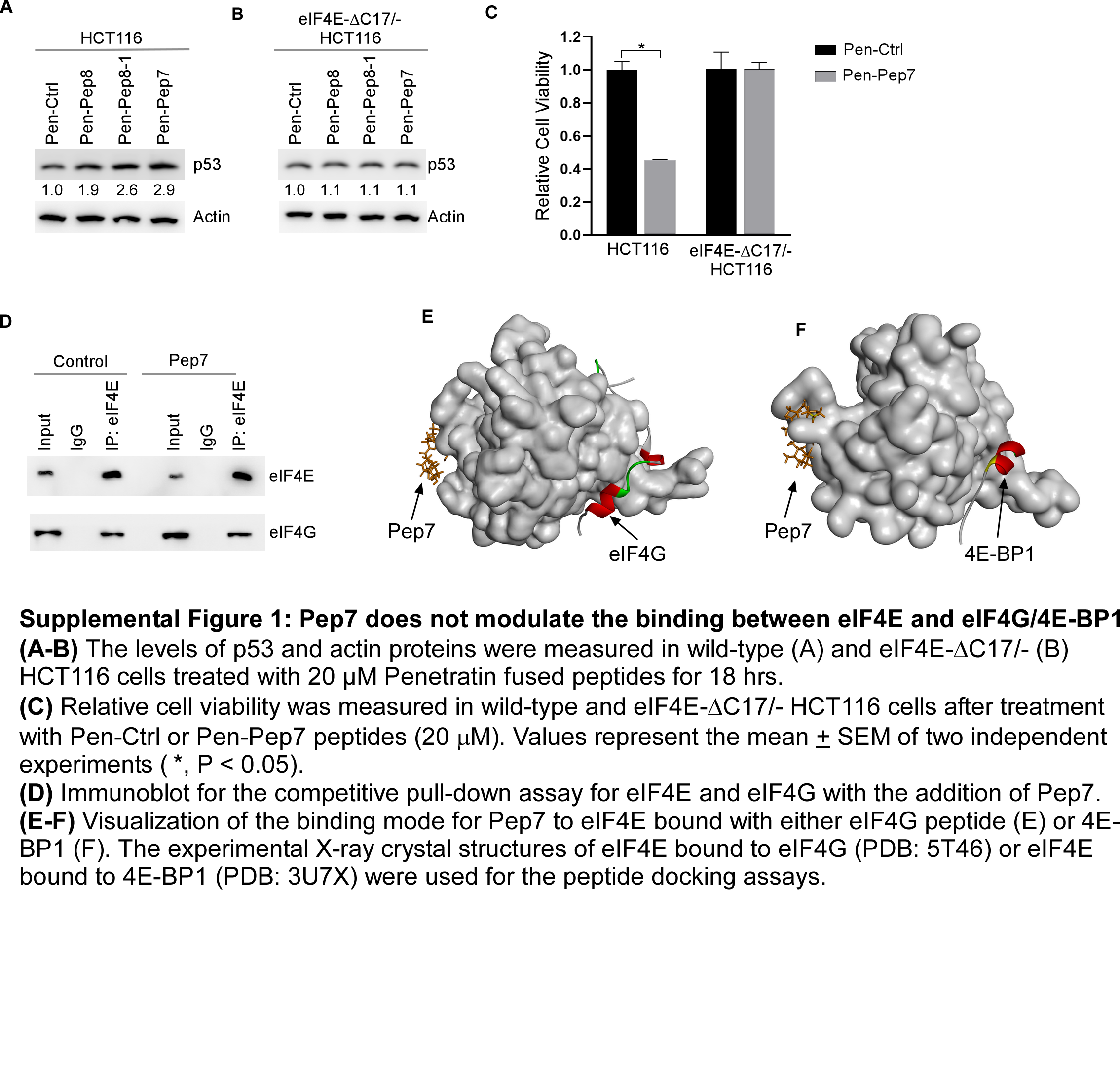

Supplement: Supplementary file 1 [file Image_1.tif]

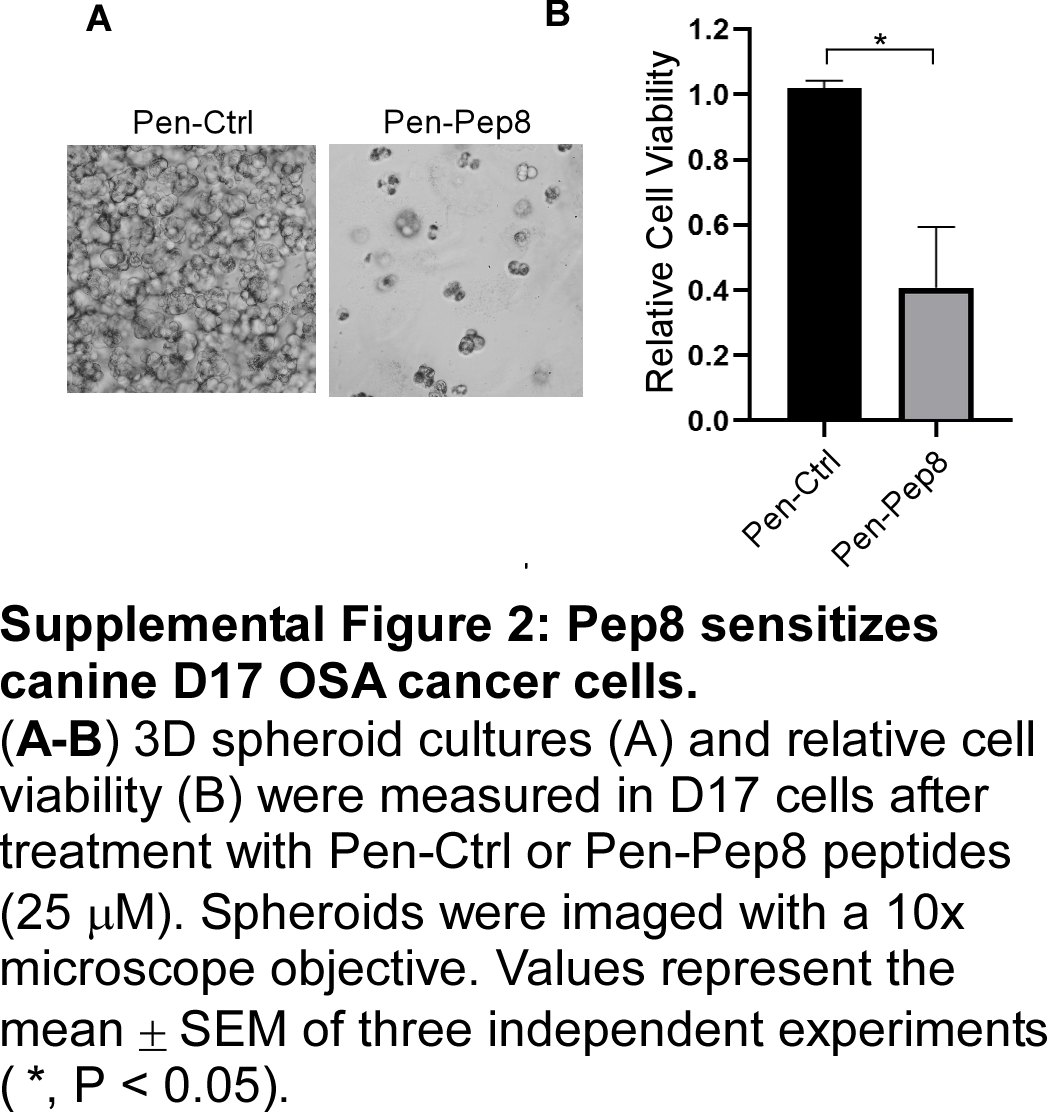

Supplement: Supplementary file 2 [file Image_2.tif]

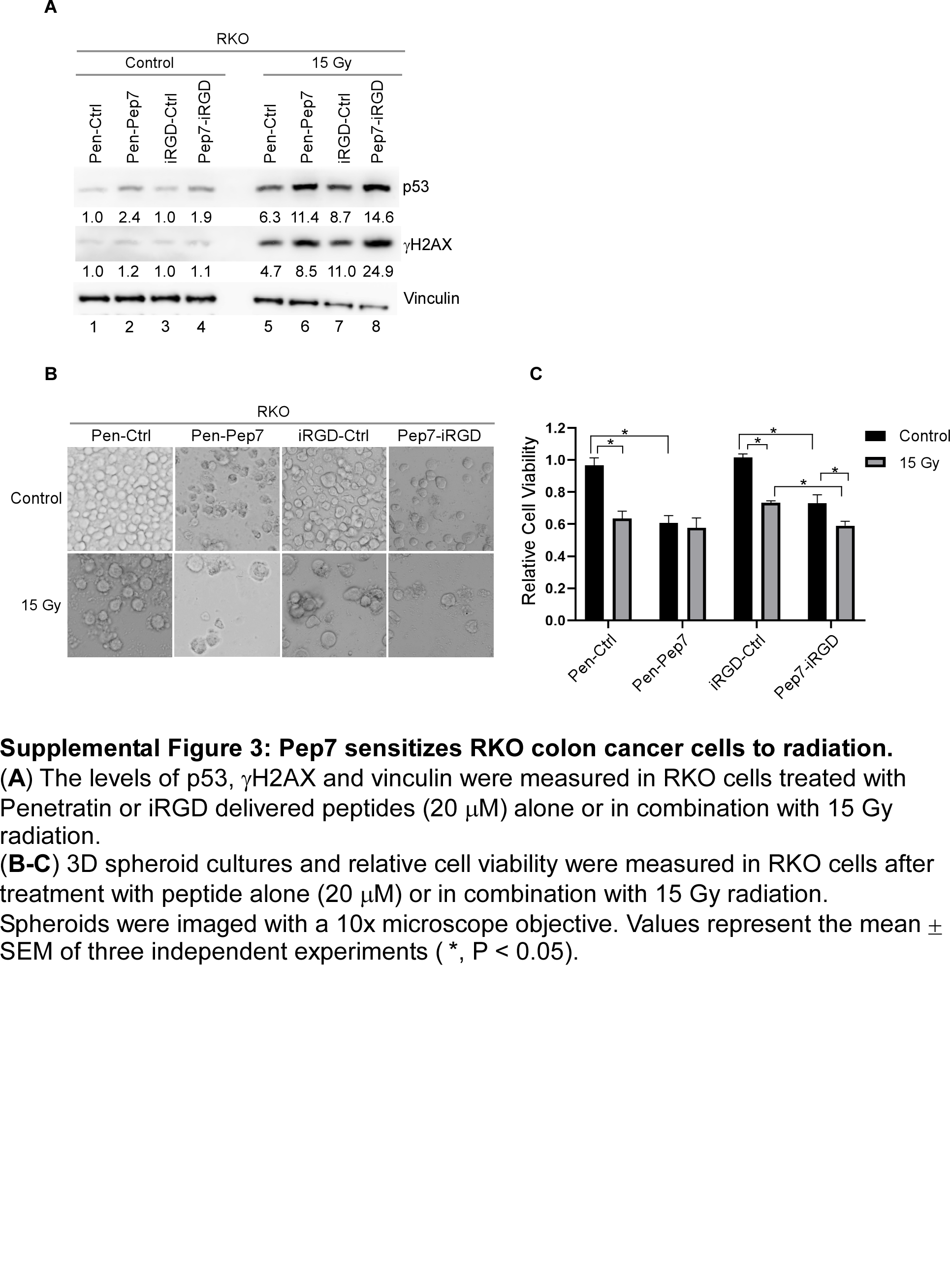

Supplement: Supplementary file 3 [file Image_3.tif]
